# The *Bos taurus* maternal microbiome: Role in determining the progeny early-life upper respiratory tract microbiome and health

**DOI:** 10.1371/journal.pone.0208014

**Published:** 2019-03-06

**Authors:** Svetlana Ferreira Lima, Marcela Lucas de Souza Bicalho, Rodrigo Carvalho Bicalho

**Affiliations:** Department of Population Medicine and Diagnostic Sciences, College of Veterinary Medicine, Cornell University, Ithaca, New York, United States of America; University of Illinois at Urbana-Champaign, UNITED STATES

## Abstract

Natural transference of maternal microbes to the neonate, especially at birth via the vaginal canal, has recently been recognized in humans and cows; however, its microbial influence on calf health has not yet been documented. We compared the bacterial communities in vaginal and fecal samples from 81 pregnant dairy cows versus those in nasopharyngeal and fecal samples collected at 3, 14 and 35 days of life from their respective progeny. The microbiota of the calf upper respiratory tract (URT), regardless of calf age, was found to be highly similar to the maternal vaginal microbiota. Calf fecal microbiota clustered closely to the maternal fecal microbiota, progressing toward an adult-like state over the first 35 days when relative abundances of taxa were considered. Sixty-four, 65 and 87% of the detected OTUs were shared between cow and calf fecal microbiota at days 3, 14 and 35 respectively, whereas 73, 76 and 87% were shared between maternal vaginal microbiome and calf URT microbiota at days 3, 14 and 35, respectively. *Bacteroidetes*, *Ruminococcus*, *Clostridium*, and *Blautia* were the top four genera identified in maternal and calf fecal samples. *Mannheimia*, *Moraxella*, *Bacteroides*, *Streptococcus* and *Pseudomonas* were the top five genera identified in maternal vaginal and calf URT samples. *Mannheimia* was relatively more abundant in the vaginal microbiota of cows whose progeny were diagnosed with respiratory and middle ear disease. Our results indicate that maternal vaginal microbiota potentially influences the initial bacterial colonization of the calf URT, and that might have an important impact on the health of the calf respiratory tract and middle ear.

## Introduction

Our understanding of the complex ruminant microbiome and recognition of the importance of the host-microbiome interaction have significantly expanded in the last few years. Bovine microbial communities have been characterized in depth across the gastrointestinal tract [1–4] and at other anatomical sites, including the mammary gland [5, 6], uterus [7, 8], and airways [9, 10]. It has been also shown that the composition of the bovine microbiome can affect host health status [10–12] and animal performance [13]. In veterinary medicine, investigation of the bovine female microbiome as a factor that influences microbial colonization early in the life of neonatal calf was recently published by Yeoman and colleagues [4], however the maternal microbiome as a contributor to neonatal calf health and/or disease predisposition, is still underexplored.

Studies in humans unveiled the potential for transference of bacterial communities from the mother to her progeny during pregnancy [14, 15] and the vertical transference via the birth canal during delivery [16, 17]. Numerous immunological and hormonal changes during pregnancy affect maternal immune regulation, leading to an anti-inflammatory biased response. This phenomenon also affects the developing immune system of the fetus [18–20], which might facilitate perinatal microbial colonization, especially at birth, by the mother’s microbiome. Exposure to microbes early in life has been shown to influence maturation of the neonatal immune system [21, 22] and potentially its metabolic development [23, 24]. Furthermore, its disruption, by interrupting the vertical transference of bacterial organisms from the mother to the newborn (e.g., as occurs with the C-section procedure), has been shown to be associated with negative downstream consequences such as asthma and allergic disorders [25, 26].

The major cause of pre-weaning and weaning calf mortality in the U.S. dairy industry is bovine respiratory disease (BRD) [27]. According to the most recent report from the United States Department of Agriculture National Animal Health Monitoring Service (NAHMS), BRD affects approximately 12.4% of the U.S. pre-weaning calves, and leads to a mortality rate of 22.5% during the same period of life [27]. Economic losses associated with BRD in the dairy industry are linked to prevention strategies, treatment, reduction in productivity and additional labor incurred [28]. This multifactorial disorder caused by a combination of microbial pathogens [29, 30], impaired host immunity [19, 28], environmental factors [28, 31], and inadequate housing conditions [28, 31], has an even larger detrimental effect on the American beef industry, causing losses of approximately one billion dollars per year [32, 33]. Several pathogens, including viruses [19, 33] and bacteria [34, 35], comprise the BRD complex that causes pneumonia in calves and potentially death. Another relatively common disease in young dairy calves is infection of the middle ear (otitis media), which is highly correlated with pneumonia [10, 36]. The upper respiratory tract (URT) seems to be an important link between these diseases [10, 36], since the nasopharynx communicates to the middle ear and resident microbes of the URT could be a source of bacteria for the lower respiratory tract [37, 38]. Furthermore, both pneumonia and otitis media share common risk factors, and the major bacteria reportedly involved in the etiology of pneumonia are also associated with otitis media [36]. Our group recently described the changes of the URT microbiome in newborn dairy calves, and determined that high total bacterial load present in the URT of 3-day-old calves, coupled with increased abundances of the bacterial genera *Mannheimia*, *Moraxella*, and *Mycoplasma*, is a key factor in the development of pneumonia and otitis media in calves during the first 60 days of life [10].

The earliest potential source of microbial colonizers in newborn calves is the maternal microbiota, which can be acquired perinatally during passage through the birth canal and from contamination by maternal fecal microbes. Interestingly, we have previously reported *Mannheimia* genus as part of the most abundant bacteria present in the pre-partum cow vagina [39]. The fact that *Mannheimia* is a strict anaerobe, unlikely to viably persist in the environment for any significant period of time, and a bacterial genus present in the calf respiratory tract and cow’ pre-partum vaginal samples, offered referral for further assessment and investigation if the vaginal microbiota play a key role in the calf respiratory tract and middle ear health.

Therefore, in the present study, we characterized the vaginal and fecal microbiotas of Holstein dairy cows within the last week of pregnancy and compared them to the fecal and URT microbiotas of their offspring to determine 1) the microbial taxa shared between dam and calf, and 2) the potential influence of the dam pre-partum vaginal and fecal microbiotas on calf URT and fecal microbial compositions at days 3, 14, and 35 of life. Additionally, we evaluated the potential influence of the dam’s vaginal and fecal microbiota on the calf’s respiratory tract and middle ear health during the pre-weaning period.

## Materials and methods

### Ethics statement

This study was conducted on a large commercial dairy farm located near Ithaca, New York. Animal Care and Use Procedures were according to the guidelines set out in Cornell University’s Dairy Cattle Husbandry publication (no. 518). All experimental protocols using cattle were reviewed and approved by the Institutional Animal Care and Use Committee of Cornell University (Protocol number: 2013–0076 and 2011–0111).

### Study design and study population

This study was a prospective observational cohort study in which 100 pregnant Holstein dairy cows from a single farm located near Ithaca, NY, were enrolled on a weekly basis. The farm was selected because of its longstanding relationship with the Ambulatory and Production Medicine Clinic at Cornell University. Vaginal and fecal samples were collected once at a single time pre-partum (273±3 days carried calf—DCC) for each cow under investigation and processed independently, as described in the next sections. Fecal and URT swabs from their respective progenies were longitudinally collected at days 3, 14 and 35 of life. After sample exclusion—due to the following reasons—delivery of twins or male calf, stillbirth, missed sample collection due to death of the calf before the end of the study and/or problems with downstream screening (e.g. failed DNA amplification or poor sequencing quality)—samples from 162 animals (81 cow–calf pairs) were subjected to further microbiome and statistical analyses.

### Animals and facilities

#### Dry cows

Pregnant heifers and cows were housed together in free-stall barns with concrete stalls covered with rubber mattresses and bedded with dried manure solids, separated from the lactating cows. Pregnant animals were housed into two separate groups: the “far-off” group (where dry cows remained until two weeks before expected calving), and the “close-up” group (cows in their last 2 weeks before expected calving). Pregnant animals that were on stage 1 or 2 (stage 1 is define as: relaxation of the pelvic ligaments, dilatation of the cervix and distention of the teats; stage 2 is define as: delivery of the calf) of parturition were moved into the deep-bedded maternity barn, with four identical group-pens (400 m^2^ deep-bedded pens), to calve. Fresh cows were first milked within 8 hours of calving in a double 52-stall parallel milking parlor. Pre-partum cows were fed a diet with a high-fiber content and low energy density. The diet was formulated to meet or exceed the nutrient requirements for lactating Holstein cows weighing 650 kg and producing 45 kg of 3.5% fat corrected milk [40].

#### Maternity

Calves were removed from the maternity pen and placed into a newborn pen bedded with dry sawdust and heated with heating lamps, right after parturition. Colostrum from multiparous (animals that experience more than one pregnancy) and primiparous (animals that experience only one pregnancy) cows was pooled and used in the study. All calves were fed approximately 4 L of colostrum at once by an esophageal feeder (Oral Calf Feeder Bag with Probe, Jorvet) within 4 hours of birth.

#### Calf barn

Twice daily, newborn calves were transferred from the newborn pen to the calf barn. The calf barn was a greenhouse type of barn with positive ventilation and divided into 18 identical group-pens. Group-pens had a total area of 70 m^2^ and were bedded with straw on top of a thin layer of dry composted manure. Steel gates divided the group pens, and calves were allocated by birth order into each pen until the pen was completely full (a total of 25 calves per pen). All calves remained in the same pen from day 1 of life until fully weaned (approximately 65 days).

Calves were fed ad-libitum acidified non-saleable milk. The feeding system was fully automated. Briefly, the acidification was performed inside a sealed stainless-steel tank where the non-saleable cold (5°C) milk was mixed continuously with organic acid until pH 4.5 was reached. The acidified milk was kept for 72 hours inside the stainless-steel tank after the acidification process was finished. Then, the milk was directed to a smaller stainless-steel tank, which maintained the milk at a warm temperature and distributed it to the pens. To support the ad-libitum system, 6 nipples per pen were connected to the smaller tank and the acidified non-saleable milk was available from day 1 to day 55 of life, when a reduction of milk availability was initiated. All calves in this study were weaned by reducing the milk availability starting on day 55 until complete absence of acidified non-saleable milk at 65 days of life.

### Sample collection

Study cows were identified and restrained in a headlock stanchion; the perineum and vulva were cleaned with a paper towel and disinfected with a 70% ethyl alcohol solution. The lips of the vulva were opened and a sterile swab (Puritan Medical Products, Guilford, ME) was applied to a single site at the midpoint of the vaginal cavity, swirled 6 times, and then withdrawn without contamination. Each vaginal swab was immediately placed in a 2-ml microcentrifuge tube (VWR International, Radnor, PA). Fecal samples were collected subsequently to the vaginal swab by using palpation gloves that were gently introduced in the rectum of the cows. Feces were then added to a sterile falcon tube (VWR International, Radnor, PA). Both vaginal and fecal samples were kept on ice until they were transferred to the laboratory at Cornell Veterinary School and stored at -80°C.

Calf deep nasal pharyngeal swabs and fecal swabs were collected from each calf at days 3, 14, and 35 of life using a 20-cm sterile swab (Puritan Medical Products, Guilford, ME) covered by a thin sterile plastic sheath. Deep nasal pharyngeal swabs were collected as previously described [10]. Briefly, prior to sampling, the calf was appropriately restrained and a nostril was cleaned using a paper towel. Subsequently, a plastic-covered swab was inserted into the nasal cavity, the plastic sheet was broken, and the swab was exposed to the URT mucosa. The tip of the swab was placed inside a sterile plastic tube and labeled. Next, under slight restraining, fecal swabs were obtained from each calf (a sterile cotton swab was inserted approximately 5 cm in the rectum). All fecal swabs were obtained at the same time as the respective URT swabs. URT and fecal swabs were frozen at -80 ^o^C until used for extraction of bacterial DNA.

### Case definition for calf pneumonia and otitis media

Diagnosis of pneumonia and otitis media was performed by experienced farm employees trained by Cornell University veterinarians (Ambulatory and Production Medicine Department) and confirmed by one of the veterinarians of the research team. Pneumonia was defined when two or more of the following clinical signs were detected in a calf: cough, rectal temperature >39.5°C, respiratory rate >40 breaths/min, increased cranioventral lung sounds or wheezes. Otitis media was defined by observation of ear pain evidenced by head shaking, scratching or rubbing the ears, epiphora, ear droop, signs of facial nerve paralysis, with or without fever (rectal temperature >39.5°C).

Calf health status was categorized as healthy (consisting of calves that did not develop pneumonia, otitis, and pneumonia-otitis combined during the pre-weaning period) or diseased (consisting of calves that developed pneumonia, otitis, or pneumonia-otitis combined during the pre-weaning period). Calves diagnosed with pneumonia and/or otitis were treated according to the standard farm protocol (Resflor Gold, Merck Animal Health). Calves that entered the farm treatment received antibiotic treatment of florfenicol and flunixin meglumine (Resflor Gold, Merck Animal Health). Drug was administered at label dose and route of administration.

### DNA extraction

Isolation of DNA from cow vaginal swabs and calf URT and fecal swabs was performed by adding 1.5 ml of DNA/RNA-free ultra-pure water into the 2-ml microcentrifuge tubes (VWR International, Radnor, PA) that contained URT, feces or vaginal swab samples. The tubes were then vortexed for 10 minutes by using a vortex adaptor (MO BIO Laboratory Inc., Carlsbad, CA) that holds 2-ml microcentrifuge tubes horizontally. The swabs were removed from the microcentrifuge tubes and the remaining liquid was centrifuged for 5 minutes at 13,000 rpm (room temperature). The supernatant was discarded and the DNA was extracted from the pellet using a PowerSoil DNA Isolation Kit (MO BIO Laboratory Inc., Carlsbad, CA) according to the manufacturer’s recommendations.

Isolation of DNA from cow fecal samples was performed after homogenization of the feces content, and a total of 250 mg of feces solution was added directly to the PowerSoil beads tube, and DNA extraction was performed following the manufacturer’s recommendations (MO BIO Laboratory Inc., Carlsbad, CA).

DNA concentration and purity were evaluated using a NanoDrop ND-1000 spectrophotometer (NanoDrop Technologies, Rockland, DE) at wavelengths of 260 and 280 nm.

### Amplicon sequencing and bioinformatics 16S rRNA amplification

For each sample evaluated, amplification of the V4 hypervariable region of the bacterial/archaeal 16S rRNA gene was performed by using the 515F and 806R primer set according to previously described methods and optimized for the Illumina MiSeq platform [41]. PCR products were tagged with 280 different 12-bp error-correcting Golay barcodes (http://www.earthmicrobiome.org/) [42]. PCRs were carried out in triplicate 25-μl reactions using 12–300 ng of template DNA, 1X EconoTaq Plus Green Master Mix (Lucigen, Middleton, WI) and 10 μM of each primer. Thermal cycling consisted of an initial denaturing step of 94°C for 3 min followed by 30 cycles of 94°C for 45 s, 50°C for 1 min and 72°C for 90 s, and a final elongation step of 72°C for 10 min. Replicate amplicons were pooled and visualized on 1.2% agarose gels stained with 0.5 mg/ml ethidium bromide, followed by purification using a Gel PCR DNA Fragment Extraction kit (IBI Scientific, Peosta, IA) and used for downstream analysis.

Amplicon concentration and purity were evaluated using a NanoDrop ND-1000 spectrophotometer (NanoDrop Technologies, Rockland, DE) at wavelengths of 260 and 280 nm.

### Sequence processing

Aliquots of fecal, vaginal and URT amplicon samples were standardized to the same concentration and pooled into 3 different sequencing runs according to individual barcode primers for the 16S rRNA gene. Final equimolar libraries were sequenced using the MiSeq reagent kit v2 (300 cycles) on the MiSeq platform (Illumina, Inc., San Diego, CA). The generated 16S rRNA gene sequences were demultiplexed using the Quantitative Insights Into Microbial Ecology 1 (QIIME) pipeline [43] version 1.9.1-dev. Sequences were filtered for quality using established guidelines [44]. Sequences were binned into operational taxonomic units (OTUs) based on 97% identity using UCLUST [45] against the Greengenes reference database [46], May 2013 release. Low-abundance clusters were filtered and chimeric sequences were removed using USEARCH [45]. Phylogenetic trees were generated from the filtered alignment using FastTree [47]. Taxonomy was assigned using UCLUST [45] consensus taxonomy assigner, against the Greengenes reference database [46] and only full-length, high-quality reads (-r = 0) were used for analysis. Additionally, we generated an OTU table using the MiSeq Reporter Metagenomics Workflow. The MiSeq Reporter classification is based on the Greengenes database, and the output of this workflow is a classification of reads at multiple taxonomic levels: kingdom, phylum, class, order, family, genus and species.

### Diversity measurements and Unifrac clustering for Principal coordinates analysis (PCoA)

To calculate how many categories of taxa or lineages were detected in each individual sample and how those taxa or lineages were distributed within samples from dam fecal and vaginal samples, and from calf fecal and URT samples, Chao1 richness [48] and Shannon diversity [49] indexes were generated using the QIIME 1 pipeline. To account for uneven sequencing depths across samples, all sample libraries were rarefied to an equal depth of 11,663 sequences before calculating the Shannon and Chao1 indexes.

To determine how taxa and or lineages were related within and between dam microbial communities and calf microbial communities, both phylogenetic unweighted and weighted UniFrac distances matrixes were generated in QIIME [50]. UniFrac distances are based on the fraction of the branch length shared between communities within a phylogenetic tree constructed from the 16S rRNA gene sequences of all groups evaluated herein (dam vaginal and fecal microbiota, and calf fecal and URT microbiota). Unweighted UniFrac is a qualitative phylogenetic metric based on the presence or absence of bacteria, whereas weighted UniFrac is a quantitative phylogenetic metric incorporating bacterial relative abundance. Principal coordinates were computed from the calculated UniFrac distance matrixes to compress dimensionality into three-dimensional principal coordinate analysis (PCoA) plots created by the “beta_diversity_through_plots.py” script in QIIME 1 and visualized by EMPeror [51]. To account for uneven sequencing depth across samples, all sample libraries were rarefied to an equal depth of 11,663 sequences before estimating the unweighted and weighted UniFrac distance matrixes.

### Statistical analysis

Alpha diversity, Chao 1 richness and Shannon diversity indexes were calculated using QIIME 1. These diversity indexes were compared within and between dam and calf microbiotas using ANOVA in JMP Pro 12 (SAS Institute Inc.). Tukey-Kramer test was used to adjust for multiple comparisons.

The OTU table obtained from bioinformatics analysis were used to describe the relative abundances of bacterial phyla and genera within the dam and calf samples. Each value obtained indicates the percentage relative frequency of reads with 16S rRNA genes annotated to the indicated taxonomic level. The profiles of dam vaginal and fecal microbiota, and calf URT and fecal microbiota within each time point (3, 14 and 35 days of life) are described for the most prevalent phyla and genera using the tabulate function of JMP Pro 12 (SAS Institute Inc.). Relative abundance of bacterial phyla is presented in a stacked chart, and relative abundance of bacterial genera is presented in a pie chart.

Differences between microbial communities (beta diversity) based on phylogenetic information visualized on the PCoA plots were calculated with analysis of similarities (ANOSIM), a non-parametric statistical method [52], in QIIME 1. ANOSIM with 999 permutations was used in this procedure to test for statistically significant differences between sample groups based on UniFrac distance matrixes. The values of the ANOSIM statistic R indicate the degree of separation across communities: R values closest to 1 suggest dissimilarity between groups, whereas R values closest to 0 suggest similarity between groups. The percentage of time that the actual R surpassed the permutation-derived R′ value is the p-value for the actual R statistic [52, 53]. In the present study, a *p-*value ≤ 0.05 was considered significant.

The similarity of bacterial community composition was evaluated by using Venn diagrams (VennDiagram package under RStudio software version 0.99.903; RStudio, Inc) for graphical descriptions of unique and shared OTUs between and within dam and calf body sites. The average of shared OTU counts was analyzed by a general linear model (ANOVA) adjusted through the Tukey-Kramer multiple comparison correction in JMP Pro 12 (SAS Institute Inc.). After defining a core microbiome across dam and calf communities, we investigated which bacterial taxa mostly accounted for similarities between groups, based on rank abundances created for each group. Heatmaps were generated to graphically represent the relative distributions of the most common bacterial genera found in the core microbiomes of the dam and calf feces groups or the dam vaginal and calf URT groups.

Repeated measures ANOVA followed by pair-wise comparison of mean levels with Tukey–Kramer test was used to compare the mean relative abundance (MRA) of bacterial genera among disease statuses (otitis, pneumonia, and pneumonia-otitis combined) and day of data collection (days 3, 14, and 35). Differences with a value of *P* ≤ 0.05 were considered significant.

To investigate whether the microbial interactions found between the dam vaginal and fecal microbial communities and the calf fecal and URT microbial communities play a role in controlling the fate of infection in calves, the relative abundances of the most common bacterial genera detected in at least 80% of dam vaginal samples and dam fecal samples were used as covariates in a discriminant analysis model built in JMP Pro 12 (SAS Institute Inc.). Discriminant analysis predicts membership in a group or category based on observed values of several continuous variables (bacterial relative abundance). Total canonical structure values, an output of the discriminant analysis that represents the correlation between the canonical variables and the covariates, were used to create the screening graphic.

Each bacterial taxon previously selected by the screening model were submitted to a two-sided Welch’s t-test followed by the Benjamini-Hochberg false discovery rate (FDR) using STAMP v. 2.1.3 [54], to examine the mean relative abundance and mean significant difference between the microbes of the vaginal microbiota of dams whose calves developed pneumonia and/or otitis (disease) from the microbiota of dams whose calves did not develop pneumonia and/or otitis (healthy). Differences with a value of *P* ≤ 0.05 were considered significant.

## Results

### Descriptive statistics

The incidences of pneumonia, otitis, and pneumonia-otitis combined in the studied calves during the pre-weaning period were 19.7% (n = 16), 34.5% (n = 28), and 6.2% (n = 5), respectively; 39.5% (n = 32) were considered healthy. The average age at first diagnosis was 23.6 (standard error of the mean, SEM = ±1.4) days for pneumonia, 24.2 (SEM = ±1.5) days for otitis, and 23.4 (SEM = ±1.1) days for pneumonia-otitis combined. Within our study cows, 44 (54.3%) were primiparous and 37 (45.7%) were multiparous.

### Sequencing results

The total post-quality-control number of sequences (sequences were filtered for size, quality, and for the presence of chimeras) used in the study was 53,564,884. The average coverage of sequences per sample was 83,434 (median = 69,134 sequences) with a SD of 51,255.

### Characterization of microbial communities

The alpha diversity in dam and calf samples was determined by Chao 1 and Shannon indices and is depicted in Fig A in S1 File. The Chao 1 richness index (number of different species) was significantly different between the dam fecal and vaginal microbiotas (Fig A1 in S1 File). No significant difference was found among the calf fecal microbiotas sampled at the different time point (days 3, 14 and 35 of life; Fig A1 in S1 File). Additionally, comparisons between calf URT samples showed them to be significantly richer at day 3 versus day 14, but not at day 35. Calf feces, regardless of age, consistently had the lowest richness index of all the samples collected from both dams and calves. However, an effect of calf age was observed for the Shannon diversity, which increased with neonatal age (*P*-value < 0.05; Fig A2 in S1 File). The calf URT samples showed significantly higher diversity on day 3 compared to day 14, followed by a further significant increase (*P*-value < 0.05) at day 35 compared to day 14 (Fig A2 in S1 File).

**Fig 1:**
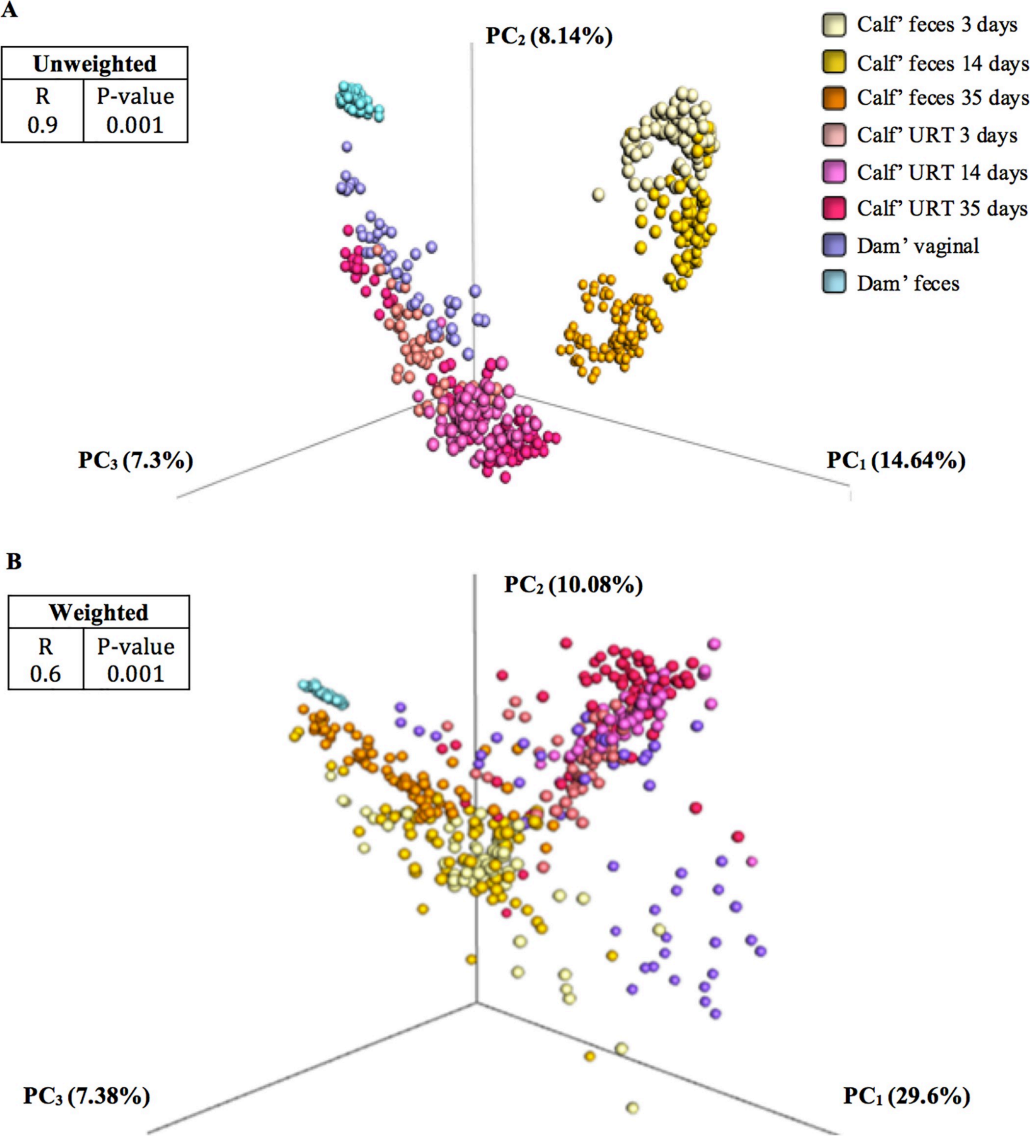
Principal coordinate analysis (PCoA) of dam fecal and vaginal microbiota, and calf fecal and upper respiratory tract (URT) microbiota at 3, 14 and 35 days of life based on UniFrac distances matrices. The variance explained by each PCoA is given in parentheses. Each point corresponds to a microbial community colored according to each type of sample (dam fecal and vaginal; calf fecal and URT) and days of life (days 3, 14 and 35 of life). Comparisons of the UniFrac metrics are depicted in a) PCoA with unweighted UniFrac, performed on presence/absence and b) PCoA with weighted UniFrac, incorporating OTU abundances. The R statistic and *P*-value for differential clustering as assessed by the ANOSIM test, based on 999 permutations, are shown in the inset. The test statistic R can range from 1 to 0. An R value close to 1 suggests dissimilarity between groups, whereas an R value close to 0 suggests similarity between groups.

The beta diversity analysis, which measures the level of similarities between samples as a function of microbial composition, was undertaken by using UniFrac distance matrixes and allowed us to evaluate the potential impact of the dam microbiota on the early calf microbiota. When combining data from all sample types (dam vaginal swabs and feces; calf feces and URT swabs over time), the samples clustered primarily by the presence and absence of taxa (Fig 1A; unweighted UniFrac R = 0.9, *P*-value = 0.001). This was not the case when OTU abundance was taken in consideration (Fig 1B; weighted UniFrac R = 0.6, *P*-value = 0.001), which would suggest that the dissimilarities between the sample types were more accounted for by the presence or absence of OTUs than by their relative abundances.

The dam vaginal microbiota was found to be highly similar to the calf URT microbiota regardless of calf age, which is clearly illustrated by the unweighted UniFrac PCoA plot (Table 1; R = 0. 37, *P*-value = 0.001). The differences (dissimilarity) in microbial composition between the dam vaginal and calf URT microbiotas account for only 37% of the variation between both microbiotas, indicating that the compositions of the dam vaginal and calf URT microbial communities overlapped by 63%. The level of similarity between the dam vaginal and the calf fecal microbiotas was much smaller (Table 1; unweighted UniFrac distance: R = 0. 72, *P*-value = 0.001 weighted UniFrac distance: R = 0. 91, *P*-value = 0.001), differing in composition by 72% (weighted UniFrac) and 91% (unweighted UniFrac). Nevertheless, when the bacterial taxa were plotted in the same PCoA space, based on the weighted average of the PCoA coordinates of all samples, where the weights are the relative abundances of the taxon in the samples, the dam fecal microbiota clustered closely to the calf fecal microbiota (Fig 1B and Table 1). Age was especially important, with a progression toward an adult-like state over the first 35 days of life (Fig 1B and Table 1).

**Table 1:** Analysis of similarities (ANOSIM) results for microbiota composition compared between sample types on weighted and unweighted UniFrac distance beta diversity. The test statistic R can range from 1 to 0. An R value close to 1 suggests dissimilarity between groups, whereas an R close to 0 suggests similarity between groups. Significance of the R statistic was determined by permuting group membership 999 times. When more than two groups were compared, in the case of calf feces and calf URT (upper respiratory tract), the samples were from all three age-groups (3, 14 and 35 days of life).

UniFrac distance	ANOSIM	
	R	*P*-value
*Dam feces*		
**Unweighted**		
Dam feces vs. Calf feces	0.92	0.001
Dam feces vs. Calf feces 35 days	0.99	0.001
Dam feces vs. Calf URT	0.72	0.001
**Weighted**		
Dam feces vs. Calf feces	0.61	0.001
Dam feces vs. Calf feces 35 days	0.39	0.001
Dam feces vs. Calf URT	0.93	0.001
*Dam vaginal*		
**Unweighted**		
Dam vaginal vs. Calf URT	0.37	0.001
Dam vaginal vs. Calf feces	0.72	0.001
**Weighted**		
Dam vaginal vs. Calf URT	0.36	0.001
Dam vaginal vs. Calf feces	0.91	0.001
*Calf*		
**Unweighted**		
Calf feces vs. Calf URT	0.65	0.001
**Weighted**		
Calf feces vs. Calf URT	0.43	0.001

Analysis of the effect of parity (primiparous vs. multiparous) on the matrixes is depicted in Fig B of S1 File. No dissimilarities were detected when the vaginal and fecal microbiotas of primiparous cows were compared to those of multiparous cows, regardless of the presence/absence of taxa (Fig B1 in S1 File, unweighted UniFrac distance; R = 0.09, *P*-value = 0.005) or relative abundance of the taxa (Fig B2 in S1 File, weighted UniFrac distance; R = 0.1, *P*-value = 0.003). A low degree of overlap was observed in bacterial community composition between the dam fecal and vaginal samples, which overlapped by 33% (Fig C in S1 File, unweighted UniFrac distance; R = 0.66, *P*-value = 0.001) and 34% (Fig C in S1 File, weighted UniFrac distance; R = 0.65, *P*-value = 0.001). Additionally, we found strong clustering between the adult fecal microbiota samples, suggesting that there is little variation in dam fecal microbiota (Fig C1 in S1 File).

Dam fecal and vaginal microbial composition at the phylum and genus levels are depicted in Fig D of S1 File. The most common genera detected in fecal samples of calves at all ages (days 3,14 and 35) are described in Figs E1, E2, and E3 of S1 File. The most common genera detected in URT samples of calves at all ages (days 3,14 and 35) are described in Figs E4, E5, and E6 of S1 File.

### The core microbiome framework within microbial communities

**Fig 2:**
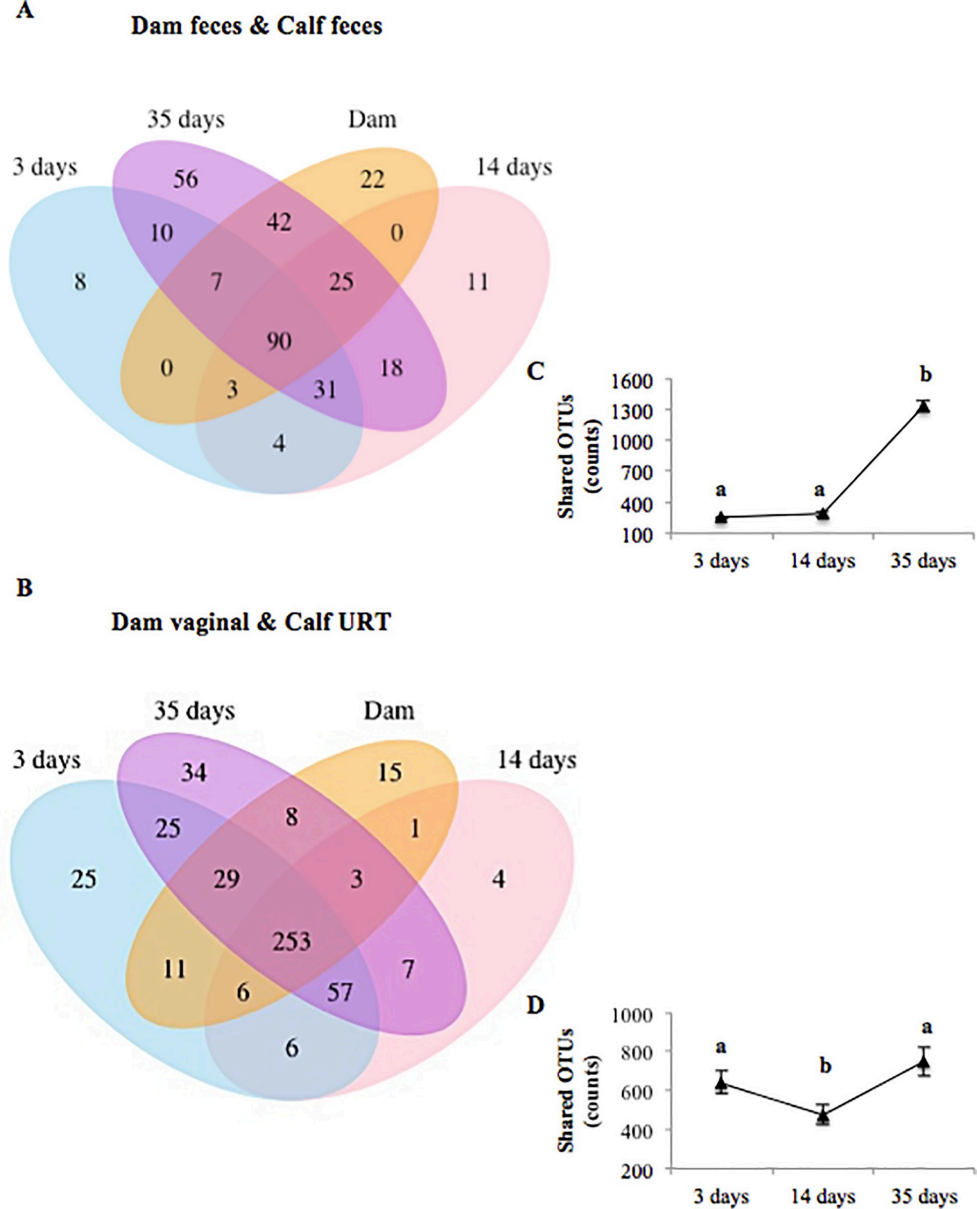
Venn diagram showing the numbers of unique and shared OTUs between dam feces and calf feces over time (3, 14 and 35 days of life) (A) as well as between dam vaginal and calf upper respiratory tract (URT) over time (B). Line graphs showing shared OTU counts between dam feces and calf feces (C) as well as between dam vaginal and calf URT over time (D). OTUs were defined at 97% sequence similarity. Error bars represent the 95% confidence interval. ^a,b^ different superscripts represent a significant difference (*P* < 0.05).

Quantification of the types and numbers of shared OTUs allowed us to evaluate the microbial relatedness among dams and calves. The Venn diagram showed that 90 OTUs were shared between the dam and calf fecal microbiotas, whereas 253 OTUs were shared by dam vaginal and calf URT microbiotas regardless of calf’ days of life (Fig 2A and 2B, respectively). Sixty four, 65 and 60% of the detected OTUs were shared between maternal and calf fecal microbiota at days 3, 14 and 35 of life respectively, whereas 73, 76 and 87% were shared between the maternal vaginal microbiota and the calf URT microbiota at days 3, 14 and 35 of life, respectively. Following the same trend that we detected in the alpha diversity analysis, the number of OTUs (counts) shared by the dam and calf fecal microbiotas increased significantly over time (Fig 2C; *P*-value < 0.05), with the number at day 35 (post-birth) being significantly higher compared to days 3 and 14. The number of shared taxa detected across the dam vaginal and calf URT microbiotas fell significantly between day 3 and day 14 (*P*-value < 0.05), but then increased significantly between days 14 and 35 (*P*-value < 0.05; Fig 2D)

**Fig 3:**
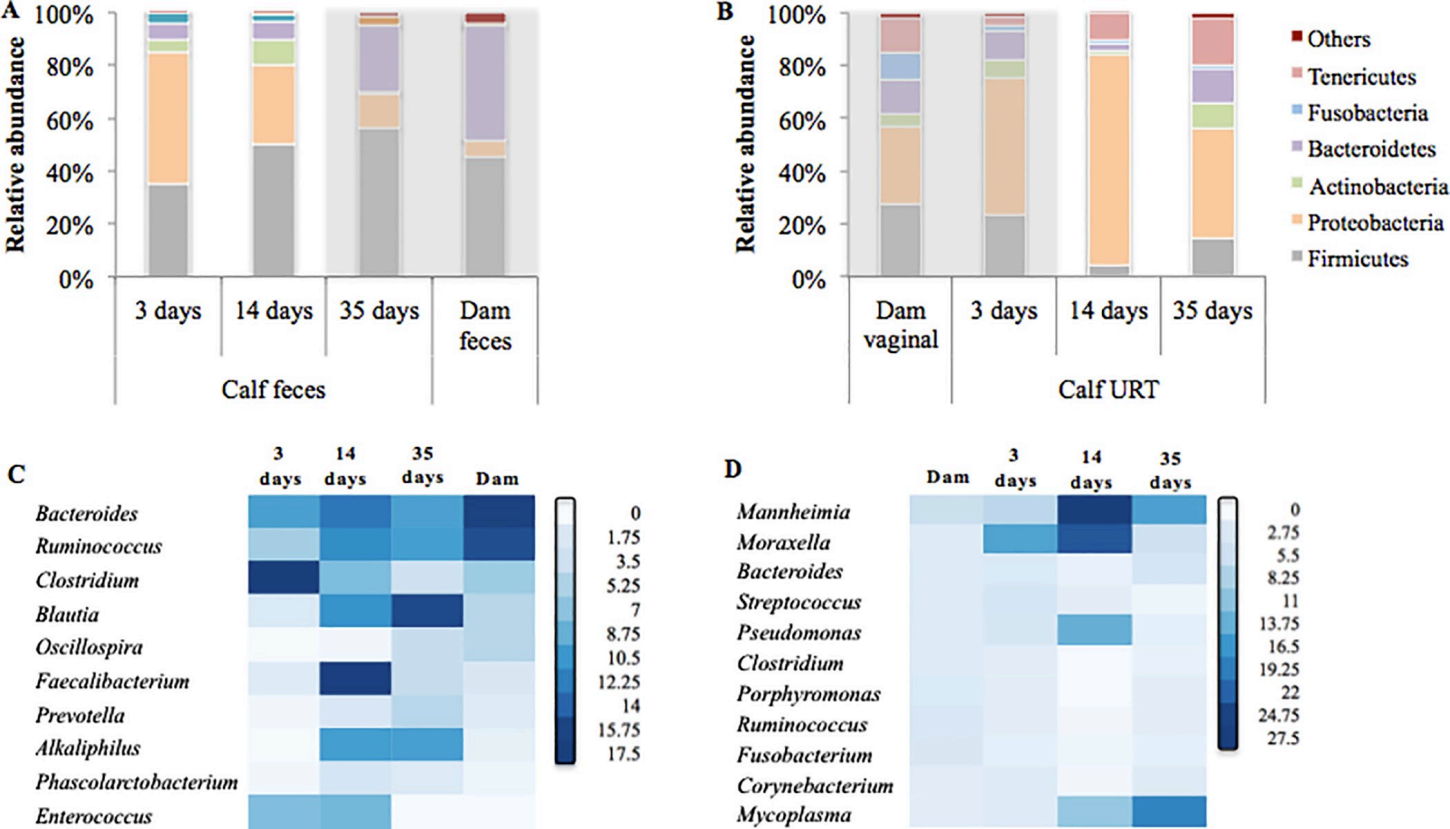
Microbial composition at the phylum level detected in dam and calf feces (A) as well as in dam vaginal and calf upper respiratory tract (URT) samples (B). The lower panels depict the 10 most common genera detected in the core microbiome of dam and calf feces (C) as well as dam vaginal and calf URT samples (D). The core microbiome was categorized as being the bacterial genera detected in all samples of dam and calf feces (C) or dam vaginal and calf URT (D). Each square in the heatmap represents the abundance level of a single category. Small relative abundance values are white, progressing to higher values as dark blue.

The dominant bacterial phyla identified in the dam vaginal and fecal microbiotas and calf fecal and URT microbiotas are illustrated in Fig 3A and 3B. At the phylum level, the fecal microbiotas of dams and 35-day-old calves were dominated by Firmicutes (45.3%, SEM = ± 2.4 and 56.3% ± 18.7, respectively) and Bacteroidetes (43.1% ± 3.1 and 25.3% ± 15.5, respectively; Fig 3A). The most abundant phyla present in the dam vaginal microbiota and the calf URT microbiota at day 3 of life were: Proteobacteia (29.83% ± 22.3 and 52.1% ± 18.8, respectively); Firmicutes (27.2% ± 15.7 and 23% ± 9.6, respectively); Bacteroidetes (12.4% ± 9.1 and 11.4.3% ± 7.3, respectively; and Tenericutes (12.8% ± 12.0 and 3.4% ± 2.4); Fig 3B).

The core microbiota genera found in the dam and calf fecal microbiotas as well as in the dam vaginal and URT microbiotas are depicted in Fig 3C and 3D. *Bacteroidetes*, *Ruminococcus*, *Clostridium* and *Blautia* were the top four genera identified in the dam and calf fecal samples irrespective of calf age (Fig 3C). *Mannheimia*, *Moraxella*, *Bacteroides*, *Streptococcus* and *Pseudomonas* were the top five genera identified within the most abundant shared bacterial genera in the dam vaginal and calf URT samples across all days of calf life (Fig 3D).

### The effect of dam microbiota on calf health

**Fig 4:**
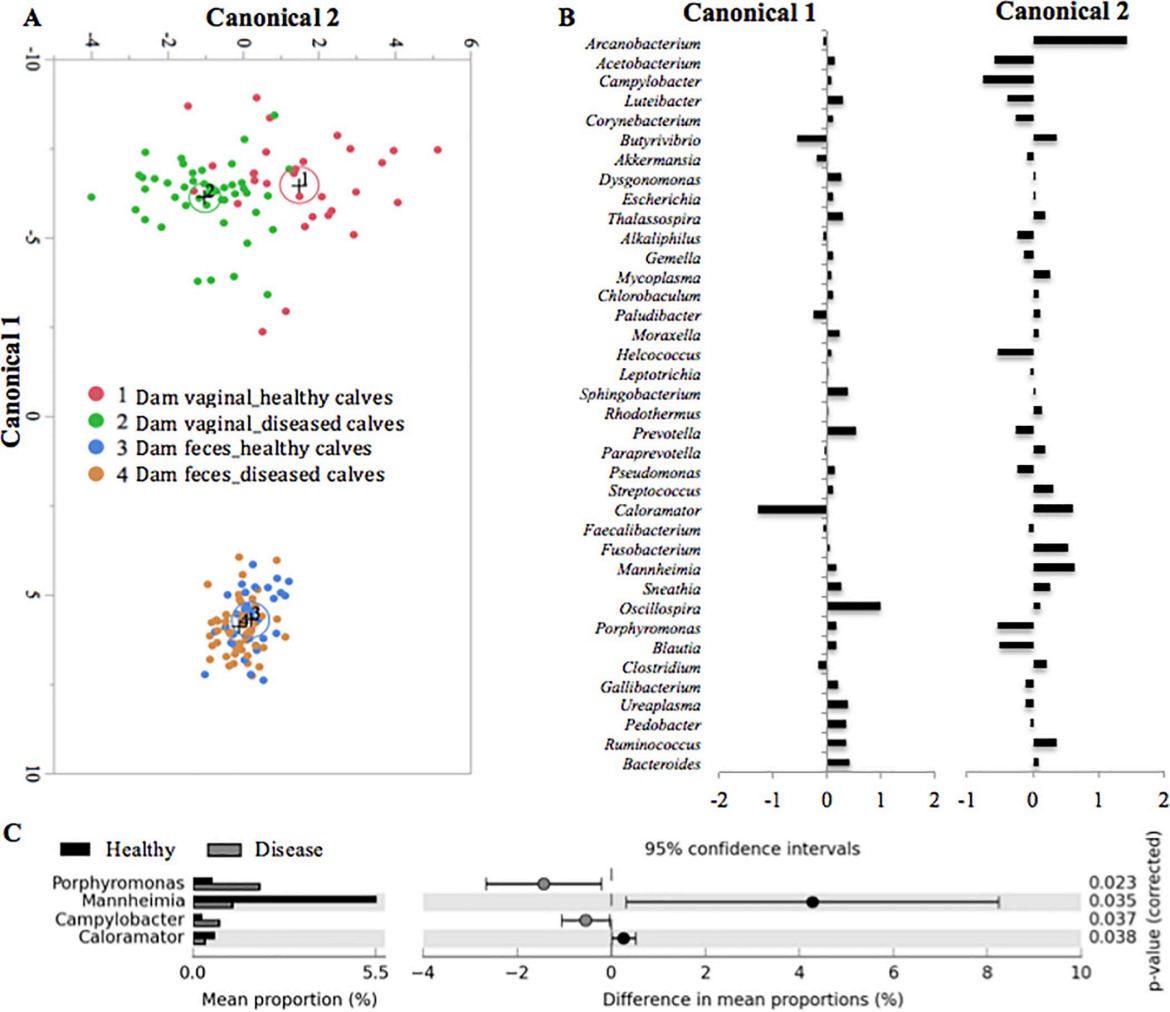
Discriminant analysis of dam feces and vaginal microbiotas according to calf upper respiratory tract (URT) health status (healthy calves, or calves that developed pneumonia, otitis, or both diseases combined during the pre-weaning period, termed ‘diseased calves’). The 40 most common shared bacterial genera were added to the discriminant procedure. The ellipses represent the 95% confidence region that contains the true mean of the group, and a plus symbol indicates the center (centroid) of each group (A). Differences in the dam microbial profiles for each health group and sample type detected in the discriminant analysis are illustrated by Canonicals 1 and 2 (B). Detailed bacterial genera differences between dam vaginal microbial groups based on calf health status are illustrated by the x-axis values (C). Welch’s test with Benjamin-Hochberg FDR correction was applied on these datasets (C). The results were filtered using a *P*-value of 0.05 and an effective size of 0.5 threshold in STAMP (C).

We evaluated whether there was a unique microbiota of maternal fecal and vaginal bacterial communities shared by all dairy cows in this study that was associated with health of the respiratory tract and/or middle ear of calves (healthy vs. pneumonia and/or otitis) during the pre-weaning period. A discriminant analysis based on dam sample type and calf health is illustrated in Fig 4A and was used to identify the most important maternal bacteria involved in the development of disease (pneumonia and/or otitis) in the neonatal calves. The discriminant function is used to calculate canonical scores that describe the separation between groups. The differences between dam fecal and vaginal microbiota as a function of offspring health status are depicted in the canonical scores 1 and 2 (Fig 4B). Fig 4C illustrates differential representation by mean proportion in the dam vaginal bacterial genera that were statistically significant different based on their progeny URT health (*P* -value < 0.05 and an effective size of 0.5 threshold in STAMP). The genera *Porphyromonas*, *Campylobacter*, were relatively more abundant in the vaginal microbiota of dams whose progeny developed URT disease, and *Mannheimia* and *Caloramator* was relatively more abundant in the vaginal microbiota of dams whose progeny did not develop URT disease (Fig 4C).

The mean relative abundance of *Mannheimia* according to calf age and health status is depicted in Fig F of S1 File. All calves regardless of age showed an increase in *Mannheimia* abundance from day 3 to 14, however within samples collected at day 14, the relative abundance of *Mannheimia* was significantly high in animals that eventually developed pneumonia than calves that remained healthy throughout the pre-weaning period (Fig F in S1 File). On the other hand, no significant differences in the MRA of *Campylobacter*, *Caloramator* and *Porphyromonas* was observed, when calf health statuses were compared, regardless of calf age (Fig G in S1 File).

## Discussion

In the present study, culture-independent technique was used to evaluate the influence of the maternal vaginal and fecal microbiotas on the microbiota and health of the neonatal calves. Our results indicate that the dam vaginal microbiota is a potential source of microbes for the URT of the newborn calf (at day 3 of life), and its influence seems to persist, at least until day 35 of life. Furthermore, a total of 253 taxa were shared between the dam vaginal and calf URT samples and *Mannheimia*, as well as, *Moraxella*, which are considered important bacteria of the BRD complex in young dairy calves [10], were part of the most prevalent bacterial genera in this shared bacterial core. Our results suggest that the health status of the calf’s respiratory tract and middle ear might be affected by mother-to-offspring transmission of bacteria. Our data also indicates that the fecal microbiota of the newborn calf differed from the fecal microbiota of the dam; however, calf fecal microbial community changed relatively quickly, such that by day 35 of life it resembled the fecal microbiota presented in the mature animal.

The structure and dynamic of fecal microbiota in the newborn calf were characterized by a gradual increasing alpha diversity over the 5-week study period, with a shift in the microbial beta diversity over the same time period. This finding was expected based on what was already known about the physiology and structure of the gut of young ruminants and the direct impact these have on gut microbial composition [55]. In addition, changes in the diet, characterized by increased consumption of solid food and reduced ingestion of milk, is typical during this initial phase. Fecal bacterial composition of pre-weaning calves from 2 weeks of life until 1 year old was recently evaluated by Dill-McFarland et al (2017), as the calves aged in their study, an increase in alpha-diversity (Shannon index) and a decrease in beta-diversity were detected [3], which corroborate with our findings. In the present study, at day 3 of life, the calf fecal microbiota was dominated by the phyla Proteobacteria and Firmicutes; however, at day 35 of life, we observed a reduction of Proteobacteria and an increase in the relative abundance of Firmicutes and Bacteroidetes, resembling the microbiota present in the dam fecal samples. The genera *Bacteroides*, *Ruminococcus*, *Clostridium* and *Blautia* were also found to be part of the 15 most abundant genera in the dam fecal microbiota and in the calf fecal microbiota at 35 days of life.

In contrast to the evident temporal changes observed in the calf fecal microbiome, we saw no dramatic differentiation of the development of calf URT microbial community, even when OTU abundance was taken into consideration using the weighted UniFrac metric. Furthermore, the calf URT at days 3 and 35 of life harbored a richer and more diverse microbial community (composed mainly of Proteobacteria, Firmicutes, Bacteroidetes and Actinobacteria) than the microbiota observed at day 14, which was dominated by members of the Proteobacteria phylum, representing 40% of the total microbial community. These findings are in agreement with data previously shown by our group [6]. This increased relative abundance of Proteobacteria was mainly driven by the increased relative abundance of the *Mannheimia* and *Moraxella* genera. Thus, the convention that respiratory-tract communities dominated by few organisms also exhibit low evenness [56] is supported by our findings of the increased relative abundance of *Mannheimia* and *Moraxella* and the lower microbial diversity index detected in the URT samples at day 14. The shift of the microbial profile detected at day 14 followed by a restoration at day 35 might be associated to what has been called the “window of susceptibility” encountered by young calves before weaning [19]. This “window of susceptibility” is a period characterized by a lower blood titer of maternal antibodies in conjunction with a still-immature calf immune system [19], conditions which might contribute to the overgrowth of particular bacterial species, resulting in the reduced URT bacterial diversity we observed at day 14 of life.

In regards to the analysis of similarity, whether calf and dam microbial communities’ sites are similar environments and contain the same species despite anatomical distance and other biological differences, calf fecal and URT samples showed dissimilar bacterial compositions when unweighted UniFrac was used. Calf fecal microbiota harbored a low diversity of microorganisms than the URT microbiota, but, by day 35 of life the calf fecal microbiota was as diverse as the microbiota observed in the URT. However, when bacterial abundance was included in the beta diversity model, it was possible to visualize some similarities between the calf fecal and URT microbial communities. These findings partially disagree with those of Dominguez-Bello et al (2010), who reported that human newborn nasopharyngeal and meconium microbial communities are essentially undifferentiated [16]. The disparate findings between the two studies might be attributed to physiological differences between humans and cattle. It is also notable that in Dominguez-Bello study, samples were collected within the first 24h of delivery (meconium), and in the present study the first fecal sample from our calves was collected 3 days of life, therefore confirming the former evidence that meconium harbored a unique microbial community that is distinct from fecal samples of the neonatal first month of life [57].

On the other hand, the composition of the maternal vaginal and fecal microbiotas collected within a week prior to parturition did not strongly cluster. Conventionally, the strong similarity between the microbiotas of the bovine reproductive and digestive tracts has been attributed to several factors, including their anatomical proximity caudally, perineal hygiene, presence of bacteria in the vagina of healthy dairy cows that are directly or indirectly associated with the cow digestive tract, and the fact that many reproductive tract diseases are caused by microorganisms found in the feces of these animals [39, 58–60]. However, this association was not evident in our study.

In the same manner, we demonstrated that the dam vaginal microbiome is similar to the calf URT microbiome; regardless of whether bacterial abundance or presence/absence is considered. Our data indicate that the bovine birth canal might be a source of microbial colonizers of the neonatal calf, and the similarity between dam vaginal samples and calf URT samples at all time points herein evaluated, indicates that the effect of the dam microbiota on the calf URT can persist. Our results corroborate to the existing literature, and also provide novel evidence to the recent investigation of the vertical transference of the bovine maternal microbiota to their progeny [4]. Our analysis identified 253 OTUs being shared between the dam vaginal and calf URT microbiomes regardless of the calf day of life sampled. Additionally, the dam vaginal microbial profile was mostly composed of Firmicutes, Proteobacteria, Bacteroidetes, and Tenericutes phyla, which is comparable to the microbiota profile detected in the URT of the neonatal calf, especially at days 3 and 35 of life. Similarities were also detected at the genera level, in which bacteria such as *Bacteroidetes*, *Mannheimia*, *Moraxella*, *Streptococcus* and *Pseudomonas* were found to be highly abundant in both the dam vaginal and calf URT samples. The occurrence and dominance of these genera within dam vaginal [39] and calf URT [10] samples are consistent with our previous studies.

Thus, in the present study, we investigate whether the dam vaginal microbiota would be a potential risk factor affecting the health of the calf respiratory tract and middle ear during the same period evaluated in our former study. Our discriminant analysis showed that the vaginal microbiota of dams whose calves went on to develop respiratory disease and/or otitis media tended to be separate from the microbiota of dams whose calves stayed healthy.

Surprisingly, and worth noting in the present study, the genus *Mannheimia* was found to be more prevalent in the vaginal microbiota of dams whose calves did not develop pneumonia and/or otitis media disease compared to the microbiota of dams whose calves did develop disease. It appears that the pre-partum higher abundance of *Mannheimia* in the vagina of dairy cows might have a protective effect on the health of the respiratory tract and middle ear of their progeny. This may reflect a natural immunization process involving delivery of dam vaginal microbes to their calves. Lee et al. (2015) showed that a robust population of dendritic cells patrols the nasal cavity in mice and humans and is poised to respond to antigens [61]. Given our interesting finding, we may suggest that antibodies generated in response to microbial colonization of the dam’s vagina shape the composition of the calf microbiota in ways beneficial to their URT health. Thus, the protective effect of the dam vaginal microbiota suggested by our study might be potentially explained by an induced antigen-specific immunity across calf nasal mucosa site.

Further studies are still needed to confirm this maternal vaginal microbiota effect on the health of their neonatal respiratory tract and middle ear during the pre-weaning period. Remembering that our study was conducted in a large commercial dairy farm, and therefore all calves that were diagnosed with respiratory disease were treated with broad-spectrum antibiotics at the day of the diagnostic, as part of the farm protocol. The impact of systemic antibiotic therapy on calf fecal microbiota was previously described [62], and most likely should affect the URT microbial composition. However, treatment with antibiotics was not done before the onset of pneumonia and otitis, therefore in the present study structure and dynamics within calf URT and fecal microbiota samples at age 3 and 14 days were not impacted by antibiotic therapy. However, calves at 35 days in the pneumonia, otitis and pneumonia-otitis combined groups were potentially not abstained from the antimicrobial effect on their URT and fecal microbiota.

## Conclusion

Our results indicate that the composition of the dam vaginal and calf URT microbial communities overlap by 63%. The dam vaginal microbiota appears to be vertically transmitted to the URT of the newborn calf (3 days of life) and to persist, at least until 35 days of life. The genera *Mannheimia*, *Streptococcus* and *Moraxella*, which are the most common bacterial genera associated with pneumonia and otitis media in calves, were among the most abundant genera in the dam vaginal microbiota. Additionally, the genus *Mannheimia* was relatively more abundant in the vaginal microbiota of dams whose progeny did not develop URT disease. Dam and calf fecal microbiotas differed in their composition by 72% (weighted UniFrac) and 91% (unweighted UniFrac). Nevertheless, when relative abundance was taken into account, the dam fecal microbiota clustered closely to the calf fecal microbiota. Age was especially important, with progression toward an adult-like state over the first 35 days of life. Together, these results provide an unprecedented understanding of the impact of the dam microbiome on the initial microbial colonization of the neonatal calf, and emphasize the need for new studies to understand the potential protective effect of the dam vaginal microbiota on calf respiratory tract and middle ear health.

## Supporting information

S1 File Supplemental 16S data. (DOCX)

S2 File Supplemental accession numbers. (XLSX)
