# Supplementary material for: The Bos taurus maternal microbiome: Role in determining the progeny early-life upper respiratory tract microbiome and health
Source: PLoS One. 2019 Mar 6;14(3):e0208014. doi: 10.1371/journal.pone.0208014 (PMC6402649; doi:10.1371/journal.pone.0208014)
Supplement: S1 File — (DOCX) [file pone.0208014.s001.docx]

**The *Bos taurus* maternal microbiome: Role in determining the progeny early-life upper respiratory tract microbiome and health**

Svetlana Ferreira Lima^1^, Marcela Lucas de Souza Bicalho^1^, Rodrigo Carvalho Bicalho^1*^

^1^Department of Population Medicine and Diagnostic Sciences, College of Veterinary Medicine, Cornell University, Ithaca, New York, USA

^*^Corresponding author: Rodrigo Carvalho Bicalho

E-mail: [rcb28@cornell.edu](mailto:rcb28@cornell.edu)

**Fig A.** Chao 1 richness (A1) and Shannon diversity (A2) indexes according to dam and calf sample types, as well as calf days of life (days 3, 14 and 35). Error bars represent the standard deviation. ^a,b,c,d,e^ different superscripts among body niches represent a significant difference (*P* < 0.05).


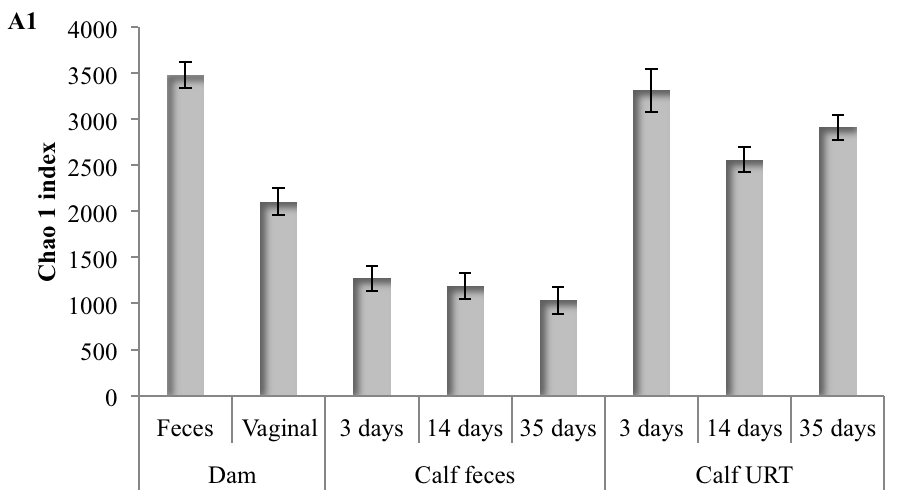


**a**

**b**

**c**

**c**

**c**

**ad**

**e**

**d**


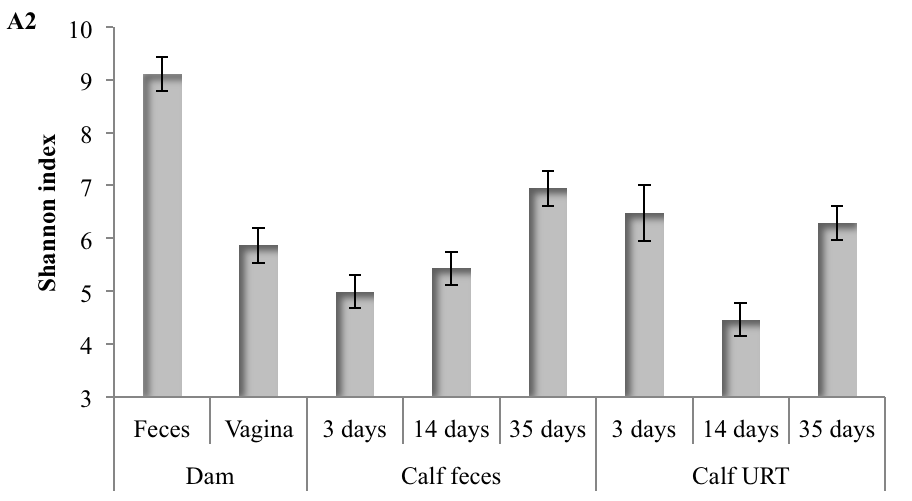


**aç**

**bd**

**cf**

**bcd**

**e**

**be**

**be**

**f**

**Fig B.** Principal coordinate analysis (PCoA) of unweighted (B1) and weighted Unifrac (B2) distances according to cow parity (primiparous and multiparous), and cow body site (gut, represented by fecal samples, and vagina). Dark purple dots represent vaginal sites of primiparous cows; light purple, vaginal sites of multiparous cows; dark blue, feces of primiparous cows; and light blue, feces of multiparous cows.

**B2**


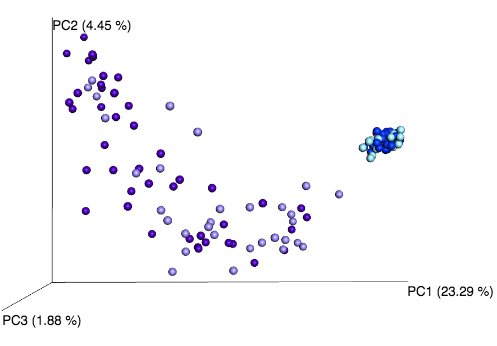


**Vaginal**

**Feces**

**B1**


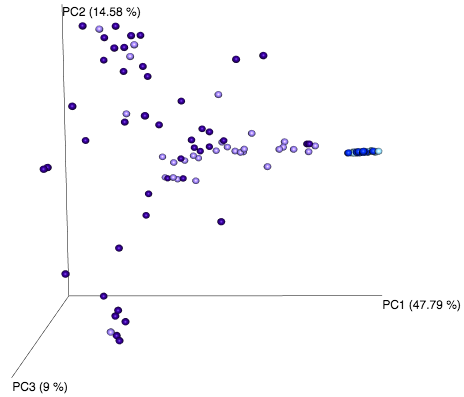


**Vaginal**

**Feces**

**Fig C.** Unweighted and weighted UniFrac distance differences between dam vaginal and feces microbiotas. An ANOSIM test was performed to compare distances between dam feces and vaginal samples and was based on 999 permutations. The test statistic R from ANOSIM can range from 1 to 0. An R value close to 1 suggests dissimilarity between groups, whereas an R value close to 0 suggests similarity between groups.


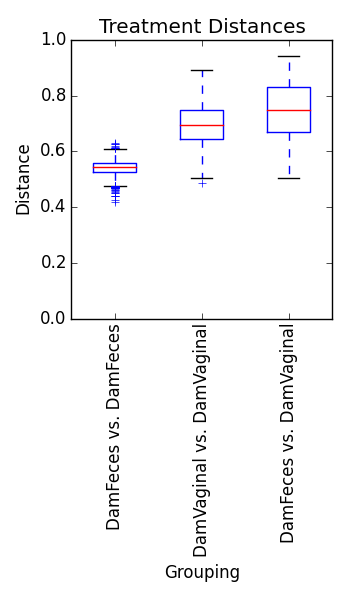

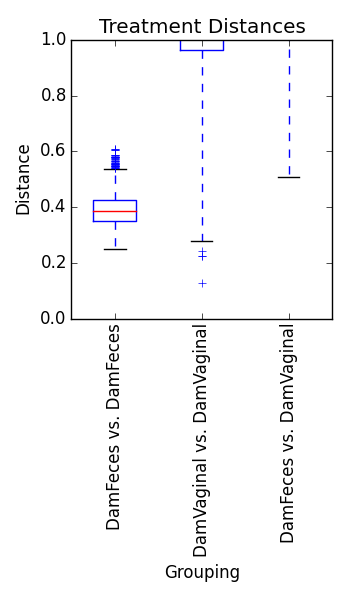


**Unweighted UniFrac**

**Weighted UniFrac**

R = 0.65

*P* - value = 0.001

R = 0.66

*P* - value = 0.001

**Fig D.** Dam fecal and vaginal microbial composition at the phylum (D1) and genus (D2 and D3) levels. Results are shown as the mean relative abundance (MRA) for the dominant phyla (D1) and for the 15 most common genera (D2 and D3) detected.

**Dam vaginal**

**Dam fecal**


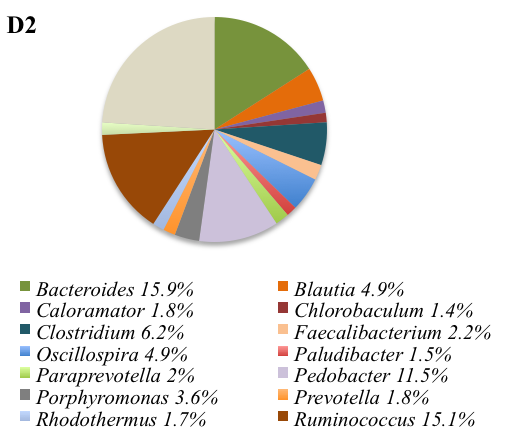


**Others**


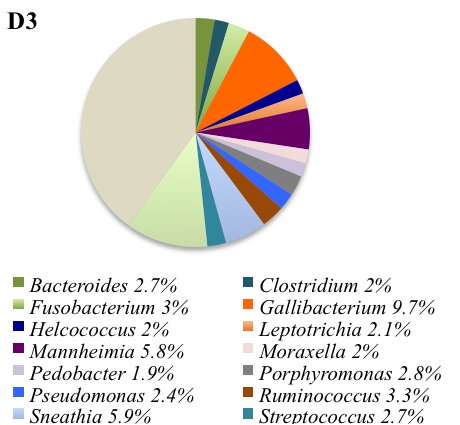


**Others**

**Fig E.** The 15 most common genera detected in calf feces at days 3 (E1), 14 (E2) and 35 (E3) of life, as well as in the calf URT at days 3 (E4), 14 (E5) and 35 (E6) of life. Results are shown as the mean relative abundance (MRA) for the dominant genera.

**35 days**

**14 days**

**3 days**


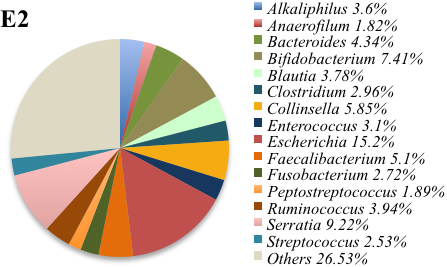


**Others**


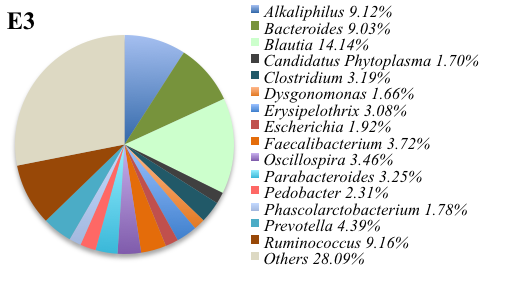


**Others**


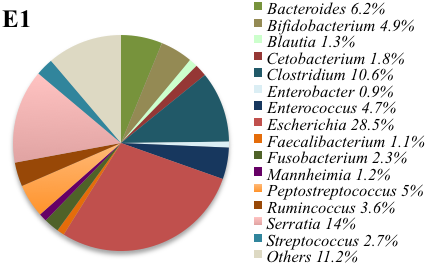


**Others**


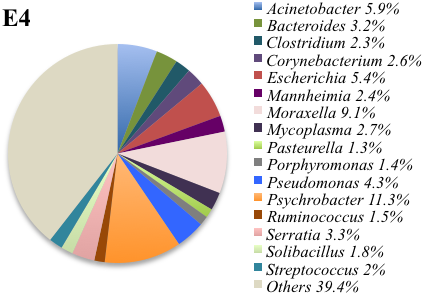


**Others**


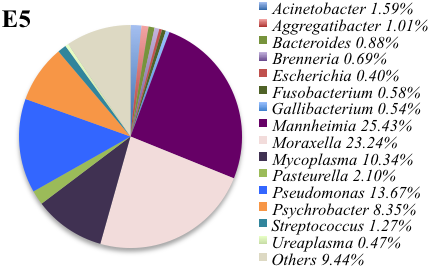


**Others**


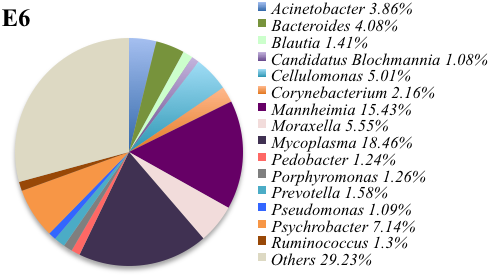


**Others**

**Fig F.** Mean relative abundance of the genus *Mannheimia* (F1) according to postnatal age at sample collection (3, 14, 35 days) and health status (healthy, otitis, pneumonia, and pneumonia-otitis combined). Error bars are positioned around the means and represent the standard error of the mean. Variables not connected by same letter are significantly different (F2, *P*-value < 0.05).

**F1**

**F2**

| **Variables** | **Tukey Significance** |
| --- | --- |
| Healthy_Day 3 | A, B, C, D |
| Healthy_Day 14 | B, C, D |
| Healthy_Day 35 | A, B, C, D |
| Pneumonia_Day 3 | A, B, D |
| Pneumonia_Day 14 | E |
| Pneumonia_Day 35 | A, B, C, D |
| Otitis_Day 3 | A, B, D, E |
| Otitis_Day 14 | D |
| Otitis_Day 35 | B, C, D, E |
| Pneumonia & Otitis_Day 3 | A, B, C, D |
| Pneumonia & Otitis_Day 14 | B, C, D, E |
| Pneumonia & Otitis_Day 35 | A, B, C, D, E |

**Fig G.** Mean relative abundance of the genera *Caloramator* (G1), *Camplylobacter* (G2) and *Porphyromonas* (G3) according to postnatal age at sample collection (3, 14, 35 days) and health status (healthy, otitis, pneumonia, and pneumonia-otitis combined). *P*-values from the repeated measure ANOVA are depicted for the variables time (day 3, 14 and 35 of life), health (healthy, otitis, pneumonia and pneumonia & otitis combined) and the health-by-time interaction. Error bars are positioned around the means and represent the standard error of the mean.

**G3**

**G2**

**G1**

Time = 0.19

Health = 0.40

Time*Health = 0.39

Time < 0.001

Health = 0.08

Time*Health = 0.43

Time < 0.001

Health = 0.20

Time*Health = 0.62
